# Spatial distribution of psychotic disorders in an urban area of France: an ecological study

**DOI:** 10.1038/srep26190

**Published:** 2016-05-18

**Authors:** Baptiste Pignon, Franck Schürhoff, Grégoire Baudin, Aziz Ferchiou, Jean-Romain Richard, Ghassen Saba, Marion Leboyer, James B. Kirkbride, Andrei Szöke

**Affiliations:** 1 AP-HP, DHU PePSY, Hôpitaux universitaires Henri-Mondor, Pôle de Psychiatrie, Créteil, 94000, France; 2 INSERM, U955, team 15, Créteil, 94000, France; 3 Fondation FondaMental, Créteil, 94000, France; 4 CHRU de Lille, Pôle de psychiatrie, Hôpital Fontan, Lille, 59000, France; 5 UPEC, Université Paris-Est, Faculté de médecine, Créteil, 94000, France; 6 Université François-Rabelais de Tours, PAV EA 2114, Tours, 37000, France; 7 Division of Psychiatry, UCL, London, W1T 7NF, UK

## Abstract

Previous analyses of neighbourhood variations of non-affective psychotic disorders (NAPD) have focused mainly on incidence. However, prevalence studies provide important insights on factors associated with disease evolution as well as for healthcare resource allocation. This study aimed to investigate the distribution of prevalent NAPD cases in an urban area in France. The number of cases in each neighbourhood was modelled as a function of potential confounders and ecological variables, namely: migrant density, economic deprivation and social fragmentation. This was modelled using statistical models of increasing complexity: frequentist models (using Poisson and negative binomial regressions), and several Bayesian models. For each model, assumptions validity were checked and compared as to how this fitted to the data, in order to test for possible spatial variation in prevalence. Data showed significant overdispersion (invalidating the Poisson regression model) and residual autocorrelation (suggesting the need to use Bayesian models). The best Bayesian model was Leroux’s model (i.e. a model with both strong correlation between neighbouring areas and weaker correlation between areas further apart), with economic deprivation as an explanatory variable (OR = 1.13, 95% CI [1.02–1.25]). In comparison with frequentist methods, the Bayesian model showed a better fit. The number of cases showed non-random spatial distribution and was linked to economic deprivation.

Ecological studies are performed at a population rather than individual level, allowing the analysis of group exposure and response without measuring individual exposure-response^1^. These studies are useful to describe the spatial distribution of diseases, and allow analyses of relationships between population characteristics and the distribution of disease cases. At a neighbourhood level, they can highlight sources of heterogeneity underlying spatial patterns, and reveal trends that may not be apparent at an individual level. Consequently, they are useful for epidemiological research and health services planning. Small area variations, in particular, are easier to interpret, and less subject to ecological bias created by the within-area heterogeneity of exposure^2^.

Most previous reports on geographical variations of non-affective psychotic disorders (NAPD)–at a “macroscopic” (i.e. between regions or countries) or at a neighbourhood level–have studied incidence rather than prevalence. Several incidence studies reported influences of various factors, such as urbanicity^3^,^4^, migrant and ethnic density^5^,^6^,^7^, social deprivation^7^,^8^,^9^ or social fragmentation^7^,^10^,^11^,^12^. Incidence studies can provide information about factors occurring before (or at) the start of the disorder, which is suggestive of causality. Thus, they are considered as the reference for epidemiological studies of disease risk factors. Prevalence estimates are influenced not only by risk factors but also by different courses of the disease. As such, they are less useful in identifying risk factors, but can provide insight on factors associated with different evolutive disease patterns, and thereby disease modifiers^13^. Such studies are therefore complementary to incidence studies. In addition, they can also provide information for the allocation of healthcare resources^14^.

Most reports on the geographical variations in NAPD prevalence have involved macroscopic variations of values^15^,^16^. Very few studies have explored prevalence rates at an ecological neighbourhood level, and even less have analysed the factors influencing their variation. For instance, Tizón compared two socially contrasted areas of Barcelona, and found a significantly higher prevalence in the lower socio-economic status (SES) area^17^. Recently, at an individual level, Termorshuizen *et al.* observed an influence of ethnic density on NAPD prevalence^18^.

Ecological studies of geographical variations of NAPD raise several methodological/statistical issues. The variance of rare count-based outcomes often exceeds the mean, and thus violates a key assumption of the Poisson regression, which is the standard statistic for count-based data. Other frequentist models could circumvent this problem, such as models using negative binomial regression. However, data relating to a set of non-overlapping spatial areal units often exhibit spatial residual autocorrelation^19^, whereby counts in neighbouring areas are more similar than counts in areas further apart^2^,^9^. This autocorrelation violates the assumption of independence of residuals and independence of variance of residuals (i.e. homoscedasticity), which are key assumptions in all frequentist methods^20^,^21^. Indeed, it means that the statistical model used to analyse the data takes into account the whole variance of the data. Frequentist models with a scale parameter could take this autocorrelation into account, but Bayesian models offer a more natural approach to simultaneously modelling spatial dependency between neighbourhoods^22^. Such models avoid these difficulties by explicitly modelling spatial auto-correlation based on an *a priori* expectation of the spatial structure (conditional autoregressive (CAR) Bayesian models)^19^,^23^. To our knowledge, only one study of prevalent cases used Bayesian methods^24^. In this study, Moreno *et al.* identified one “hotspot” (i.e. clusters of high prevalence areas) of schizophrenia. This study shows the utility of Bayesian spatial methods for geographical analyses of NAPD. However, the authors did not study the relationship with ecological variables.

The aim of the present study was to study the spatial distribution of prevalent cases of NAPD in an urban area of France, and analyse its relationship with ecological variables. To this end, we assessed validity and goodness of fit of several frequentist and Bayesian models. Based on previous studies on the distribution of incident or prevalent cases, we also included in these models three ecological/population variables: migrant density, economic deprivation and social fragmentation.

## Methods

### Catchment area and population at risk

The catchment area included two adjacent cities in the southeast of Paris (France): Créteil and Maisons-Alfort. For the enumeration of census data, the French National Institute for Statistics and Economic Studies (“INSEE”) divides cities in geographical areas (named “IRIS”). These areas are homogeneous in habitat type. Boundaries between IRIS areas are based on major natural or man-made features of the urban fabric (main roads, bodies of water, etc.)^25^. Créteil and Maisons-Alfort include 54 IRIS. One peripheral area, which was estimated to include 925 residents, designated as an area for travellers, was excluded from analyses because of difficulties in enumerating this population accurately for both NAPD cases and census data. According to the 2010 census, the 53 remaining IRIS comprise between 1223 and 4977 residents (mean: 2064, standard-deviation: 705), making a total population at-risk (i.e. 18 years old and over) of 109 397 (66 681 in Créteil and 42 716 in Maisons-Alfort)^26^.

The catchment area is a densely populated area, with 8568 inhabitants per square kilometre, with a high migrant density (migrants represent 19.8% of the population), and a high unemployment rate of 12.6%. For comparison, the larger Ile-de-France region, in which our catchment area falls, has a population density of 991 per square kilometre, 18.2% of migrants and 8.8% unemployment rates. In mainland France, the density is 99, the migrant density is 8.9% and the unemployment rate is 10.6%.

### Case finding and data collected

Two 8-week studies of the treated prevalence of NAPD (namely schizophrenia, schizophreniform disorder, schizoaffective disorder and chronic delusional disorder) took place in Créteil and Maisons-Alfort. The methods used are described in detail elsewhere^27^ and summarized below.

All physicians working in the catchment area and likely to treat patients for NAPD, namely psychiatrists and general practitioners (GPs), were contacted. During 8 weeks (in 2014 for Créteil, 2015 for Maisons-Alfort), all practitioners who agreed to participate prospectively reported on the NAPD patients that they had seen. Inclusion criteria were: 18 years old and over, meeting a diagnosis of NAPD according DSM-IV-TR (codes 295.xx, 297.x, 298.x)^28^ and receiving antipsychotic treatment prescribed during the consultation. Exclusion criteria were: psychotic symptoms caused by the effects of a substance; a general medical condition; or a mood disorder. Special attention was given to avoid patient duplications. Socio-demographic data concerning each patient, including the IRIS of residence, were collected. This prospective report was complemented by several methods estimating the number of missed cases, including leakage studies, which led to the identification of additional cases. Patients living in long-term care facilities or outside the catchment areas were excluded from the spatial analyses of the present report.

Prevalence rate ratios, indicating the ratio between actual prevalences and the expected prevalences, were calculated for each IRIS. Expected prevalences were calculated on the basis of the prevalence by age-band and gender in the overall catchment area and on the number of persons at risk by age-band and gender in each IRIS.

The relevant Regional Ethical Committee (Comité de Protection des Personnes Ile-de-France VI) examined and approved the study protocol (number 2011-A01209-32) in accordance with the Helsinki Declaration. Written consent was not requested because the Ethical Committee agreed that, for ethical reasons, it was important to preserve anonymity of the subjects. Thus, all data sent to the researchers were anonymous and patients were not in contact with the research team.

### Statistical analyses

#### Overview

Our aim was to study the spatial distribution of NAPD, thereby identifying the most appropriate statistical model, i.e. that best fitted the data. To achieve this, we modelled the number of cases in each area (dependent variable) as a function of several independent variables. We used three main statistical methods, of increasing complexity: Poisson regression, negative binomial regression and Bayesian (spatial) methods. We began with the simplest method (Poisson) and moved to more complex methods only if, based on validity tests, this proved to be necessary. For each tested statistical method, we used a forward-fitting selection method to test models with an increasing number of explanatory variables.

The independent variables were chosen among potential confounders and explanatory variables. As the number of cases in each IRIS could reflect difference from the number of at risk residents, percentage in age-bands, or gender^16^,^29^, we systematically adjusted for these factors (i.e. confounding variables). Based on previous research, we chose to study three putative explanatory variables, which are detailed below. The initial models included only the potential confounders, followed by models with an increased number of explanatory variables, which were retained only if they improved less complex models.

Methods to choose the best model, among options that differed in the number of explanatory variables for all statistical methods, as well as in in their prior assumptions for the Bayesian models, were based on the usual statistical fit diagnostics: Akaike Information Criterion (AIC) for frequentist models, and Deviance Information Criterion (DIC) for Bayesian models^30^. These statistical tools give an estimate of the model fit, penalized for complexity, such that smaller values indicate better models.

Finally, based on the best model selected in the previous steps, we calculated the values of posterior relative risk (RR), i.e smoothed risk, in order to identify areas that showed a significant increase in prevalence (“hotspots”).

#### Data used in the analyses

To adjust for age and gender, we used the procedure recommended by Guo^31^. Four age-bands were available for the denominator from the census data. Prevalences for the two genders were similar in 3 of the age-bands, and significantly different in the 25–39yr age-band. Thus, the adjustment variables included were: the proportion of residents at risk in the 5 different groups (18–24yr age-band, females 25–39yr, males 25–39yr, 40–54yr and 55yr+). The 55yr + age-band was used as a reference category and, thus, not included in the model. Finally, to account for the differences in population size between different IRIS areas, the log of the number of persons at risk was used as an offset. All denominator data came from the 2010 French national census.

Based on the previous literature concerning NAPD^10^,^16^,^32^ and from data available from the census, the independent explanatory variables included in the statistical models were measures of economic deprivation (ECON), migrant density (MIG) and social fragmentation (FRAG) for each IRIS. To calculate these variables, we used proxies derived from the most recent available census measures (i.e. 2010 census)^26^. ECON was based on the percentage of people unemployed and the proportion of households not owning (at least) one car^8^,^10^,^11^. MIG was based on the percentage of first-generation migrants (i.e. those foreign-born) and of foreigners in the area^6^,^18^. FRAG was based on the proportion of people who had lived in an IRIS for less than 2 years and the proportion of people living alone^10^. For each of this three measures, we used the composite of two standardized scores, with a mean of 0 and a standard-deviation of 1 (i.e. a Z-score), before summing them, similar to the procedure adopted by Allardyce *et al.*^10^.

#### Statistical modelling: non-spatial (frequentist) approach

The first analyses used frequentist models, i.e. models considering IRIS as randomly distributed, whereby the number of cases in each IRIS were analysed independently of location. The first model used the Poisson regression. The validity of this method requires that the mean of the dependent variable is (approximately) equal to its variance. However, in small area-level studies of rare disorders, the variance of the number of cases often exceeds the mean, defined as overdispersion^9^. Consequently, dependent variable overdispersion was tested using Dean’s test^33^.

In case of significant overdispersion, a negative binomial regression model was used instead, as recommended by Cameron et Triverdi^21^. As stated above, for these two frequentist models, we used a forward-fitting selection. We begun with cofounding variables, and then added each of the explanatory variables (ECON, MIG, FRAG) in turn. If the AIC score showed an improvement of the fitting, we chose the best model and then tested more complex models adding the remaining explanatory variables, one at a time; and so on for the third explanatory variable.

Once we found the best frequentist model (using Poisson or negative binomial regression), we used Moran’s I test to assess the existence of spatial residual autocorrelation^34^. The existence of a significant spatial autocorrelation points to the necessity of using CAR models to represent this spatial autocorrelation.

Finally, to graphically represent the quality of fitting of the best frequentist model, we mapped the ratio between fitted values from the model and actual values from the data.

#### Statistical modelling-spatial (Bayesian) approach

CAR models are specified in a Bayesian framework, where inference is based on Markov Chain Monte-Carlo (MCMC) simulation, i.e. simulation to combine the prior distribution with the data, leading to the posterior likelihood^19^. Bayesian models allow the correlated structure of random effects to be specified a priori, with CAR models based on an adjacency matrix of the areal units. Several versions of the CAR model have been developed, differing in the prior assumptions about the spatial structure of the data. To choose the spatial model that best suits the data, we compared the different models implemented in CARBayes R package. The first model is the independent model (IND), which assumes no spatial correlation and weights the risk in each area toward the overall mean. The second model is the intrinsic autoregressive (IAR) model, which weights the risk in each area by the risks in immediately adjacent areas. More complex global models, which make assumptions about the nature of the spatial random effect, include both a strong spatial correlation component (between neighbouring areas) and weaker spatial correlation (between areas further apart). Two such models are implemented in CARBayes: Besag, York and Mollié's (BYM) and Leroux’s models (for more details, see Lee^19^). Leroux’s model, particularly, includes separate parameters for overdispersion phenomenon and the strength of spatial dependence^35^. To find the best fitting Bayesian model (type of model and explanatory variables), we used the forward-fitting modelling approach, as described for frequentist approaches, and using the DIC to assess the fit.

As is standard practice with Bayesian hierarchical modelling, all models were run for several thousand “burn-in” iterations to achieve convergence. This convergence was assessed using Geweke’s method (with Z-score absolute values from Geweke diagnosis <1.96 suggesting that convergence has been achieved)^36^.

As for frequentist model, we mapped the ratio between fitted values from the model and observed values, in order to graphically represent the quality of the model.

Finally, we compared the fit of the best non-spatial and spatial models. To this end, we used two statistical criteria: the coefficient of determination (r^2^) between observed and predicted values for each IRIS, and the root mean squared deviation (RMSD). We considered a higher proportion of variance explained by the model (higher r^2^) and a smaller mean differences between predicted and observed values (smaller RMSD), as indicative of a better fit.

Once the model that best fitted the data was identified, we calculated the values of smoothed risk, as the number of cases predicted by the model (posterior fitted values) divided by the expected number of cases (based on the standardized prevalence, according to gender and age) in each area. Following Richardson *et al.*, we considered that smoothed risk that exceeded unity with a 75% probability identified raised-risk areas, with neighbourhoods having such smoothed risk being defined as “hotspots”^2^.

#### Software

For all analyses and map creations, we used the R software version 3.1.0 (http://www.R-project.org), with the CARBayes package for the spatial analyses^19^. Geweke’s method was used as implemented in the Coda package^37^.

## Results

### Descriptive statistics

462 patients treated for NAPD were identified, including 104 living outside the catchment area or in long-term care facilities; thus 358 cases were finally included in the spatial analysis (64.0% from Créteil; 36.0% from Maisons-Alfort). Most of the cases were outpatients (83.7%), being treated by psychiatrists (97.7%) in the public sector (93.3%).

**Figure 1:**
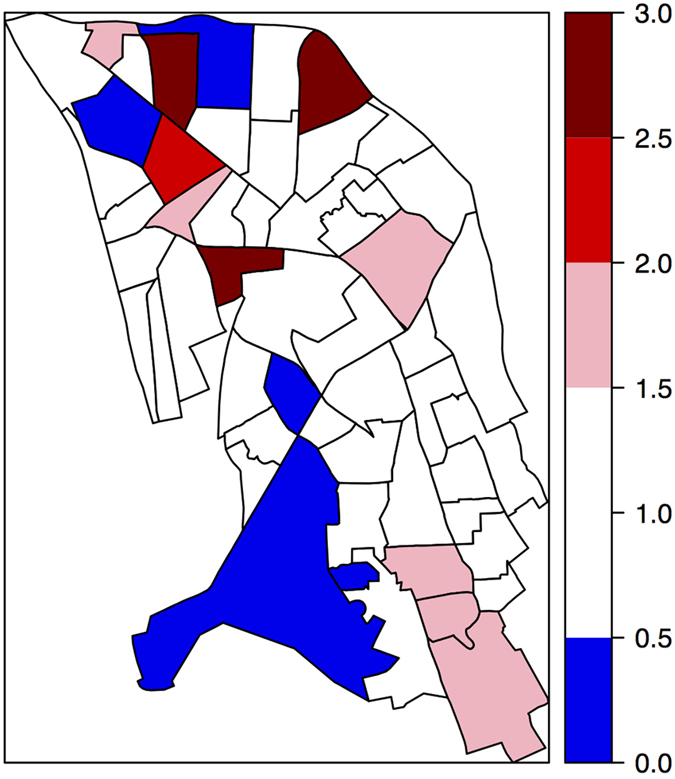
Map of prevalence rate ratios per IRIS (i.e. ratio between actual prevalences per IRIS on expected prevalences^1^ per IRIS). ^1^Expected prevalence is calculated from the prevalence by age-band and gender in the overall catchment area and the number of persons by age-band and gender at risk in each IRIS. Map created with R software (http://www.R-project.org) version 3.1.0.

Number of cases per IRIS ranged from 0 to 23 (mean number of cases per IRIS: 6.75, standard-deviation: 4.29). Numbers of cases and prevalences per gender and age-band can be found in Table 1. Figure 1 represents the map of prevalences rate ratios per IRIS.

### Statistical modelling

#### Frequentist methods: Poisson and negative binomial regression models

The best Poisson model (smallest AIC) was the one with ECON as sole explanatory variable (with more cases in more deprived areas, OR = 1.13, 95% CI [1.04–1.23], p = 0.003). However, as Dean’s test showed an overdispersion (result: 7.48 > 1.96), the model was not valid for the data.

The best negative binomial frequentist model was the one with FRAG as sole explanatory variable (non-significant association: OR = 0.89, 95% CI [0.78–1.02]), p = 0.12). The Moran test (p = 0.05) suggested that autocorrelation of residuals might be present. Thus, we decided to implement Bayesian/spatial statistical methods, and then to compare Bayesian and negative binomial frequentist best models.

A detailed description of the steps and results that led to this selection is provided in Table 2.

**Figure 2:**
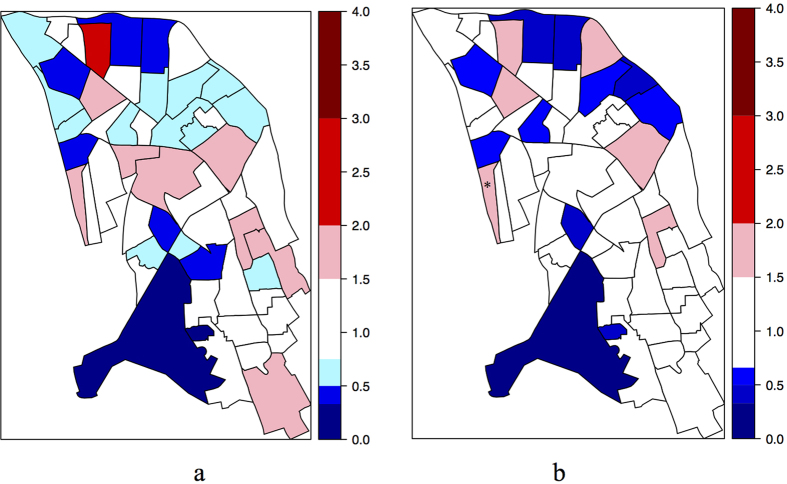
Map of ratio of observed values on best frequentist (**a**) and Bayesian (**b**), with the IRIS “hotspot” marked with an asterisk (*) models fitted values per IRIS. Map created with R software (http://www.R-project.org) version 3.1.0.

Figure 2a graphically represents the quality of fitting of the binomial model with FRAG as explanatory variable.

#### Bayesian methods

Comparison of the different Bayesian models showed that the best model (smallest DIC) was Leroux’s one with ECON as the only explanatory variable. As such, the CAR prior model that best fitted the data was characterised by both a strong correlation between neighbouring areas and a weaker correlation between areas further apart. The numbers of cases per IRIS were influenced by the economic deprivation in the small area units (with more cases in more deprived areas: OR = 1.13, 95% CI [1.02–1.25]). MIG (OR = 1.09, 95% CI [0.99–1.19]) and FRAG (OR = 0.90, 95% CI [0.78–1.04]) were not significantly associated with the number of cases per IRIS. A detailed description of the steps and results that led to this selection is provided in Table 3.

The absolute value of the Z-score (0.08) from Geweke diagnosis was lower than 1.96, showing that the model converged.

Using the 75% probability threshold to calculate smoothed risk, one hotspot was identified, being the one area in which the prevalence was significantly increased compared to that expected in the model.

Figure 2b graphically represents the quality of fitting of Leroux’s Bayesian model with ECON as explanatory variable. An asterisk marks the hotspot.

#### Comparison between frequentist and Bayesian models

Compared to the negative binomial frequentist model, Leroux’s model performed better as to the variance explained (r^2^ = 0.70, compared to 0.31) and RMSD between predicted and observed values (RMSD = 324.14, compared 667.56).

## Discussion

### Main findings

In this study, we analysed, for the first time in France, the spatial distribution of prevalent cases of treated NAPD. This was also the first study to explore the determinants of NAPD at an ecological neighbourhood level. The main findings of this analyses showed that, in this urban area, the distribution of cases of NAPD showed non-random spatial distribution and was associated with economic deprivation. Secondly, for the first time, we showed that the fit of frequentist models was weaker than that of the Bayesian models. The model that best fitted the prevalence data was Leroux’s Bayesian model, which involves a strong correlation between neighbouring areas and a weaker correlation between areas further apart. This confirms the conclusion of Moreno *et al.* as to the necessity to use Bayesian spatial models to take into account the residual autocorrelation in analyses of small area level variations^24^. It is of note that the best frequentist (non-spatial) model and the best Bayesian (spatial) model (i.e. negative binomial and Leroux’s models) led to different results. Moreover, as we identified only one hotspot on 53 areas, the variability of the prevalence was almost totally present in Leroux’s statistical model with the confounding variables (age, gender) as well as economic deprivation.

### Comparison with precedent findings

Comparing our results with previous ecological findings is difficult due to important differences between this study and previous reports, particularly in statistical methods used and explanatory variables tested.

Scully *et al.* studied neighbourhood variations of place of birth and onset of prevalent cases of psychotic disorders, finding a significant deviation from the Poisson regression model for place of onset^38^. They did not test for potential overdispersion and/or residual autocorrelation of the outcome, thereby making it difficult to compare their results to the findings of this study. Furthermore, their study comprised rural areas that were larger and had lived-in areas further apart than those in the present study. As such, their outcome was less likely to exhibit spatial autocorrelation. Finally, they studied place of onset of the disease, which is linked to incidence distribution and not to prevalence distribution.

In their spatial analysis of schizophrenia prevalence in the province of South Granada, comprising rural and urban areas, Moreno *et al.* aimed to identify hotspots of treated cases of schizophrenia. They used robust methods, namely Moran’s test which detected spatial autocorrelation of the distribution of the outcome, which was taken into account by the use of the Bayesian spatial (BYM) model. As in the present study, these authors identified one hotspot area, which was in a zone with a very low mental healthcare accessibility. In comparison to the present study, the catchment areas were larger and less tightly connected. Also the Moreno *et al.* study did not test for ecological variables that could explain spatial distribution^24^.

### Association with ecological variables

Our study is the first to model the ecological effect of economic deprivation on spatial distribution of prevalent NAPD cases. Several previous prevalence-based study findings are consistent with an association between the distribution of NAPD and economic deprivation^39^. Several individual-level hypotheses may be proposed to explain such an association. Firstly, as economic deprivation is associated with the incidence of NAPD^3^,^7^,^8^,^9^,^40^,^41^, it could explain higher prevalence in deprived areas. Social drift has been suggested as a cause of the increased number of NAPD cases in deprived areas^9^. Indeed, poorer social and cognitive functioning, characteristic of NAPD, could cause social marginalization^42^, unemployment^43^, economic deprivation, and subsequently relocation in poorer areas. Social drift is often opposed to social causation theory. However, for prevalent cases it may act in an additive way. Analyses of the migration of patients after a schizophrenia diagnosis in Quebec shows that patients are more likely to stay in, or migrate to, the most materially deprived territories^44^,^45^. Secondly, NAPD patients may experience stigma, which preclude employment, consequently increasing the risk of poverty and habituation in deprived areas^46^. In this way, stigma can heighten social isolation and social drift. Thirdly, economic deprivation may be a modifier factor. Indeed, patients living in deprived areas could have a more severe illness, including a longer duration of untreated psychosis and more severe cognitive impairment, as well as more addictive or depressive comorbidities. Such factors may contribute to lower remission rates and to an increase in the proportion of NAPD patients in these areas. Consistent with this hypothesis, an experimental study by Ellett *et al.* showed that walking in deprived urban areas can provoke paranoid thoughts in patients with persecutory delusions^47^. These issues need to be addressed in studies at an individual-level. Moreover, these considerations should not obscure implications for the allocation of health services. Our study shows that more deprived areas harbour the greatest need for psychosis care. Previous studies showing that incidence was higher in deprived areas suggested that prevention strategies have to focus on deprived areas^40^,^41^. Results of the present study suggest that higher levels of psychiatric services for psychotic disorders are required in more deprived areas.

This study showed no linkage of migrant density and social fragmentation to the spatial distribution of cases. Previous reports indicated significant associations between these factors and higher prevalence (for migrant density) and incidence (for both). One possible explanation is a lack of statistical power. However, although the association with economic deprivation was weak (OR = 1.13, 95% CI [1.02–1.25]), the statistical power of our study was sufficient to show any association. Another potential explanation could also be a selection bias. Indeed, we studied treated cases of NAPD, whilst people living in high migrant density or socially fragmented settings may have more difficulty in accessing healthcare^41^,^48^. Furthermore, as studies showing associations between social fragmentation or migrant density with incidence or prevalence measures have been conducted in different contexts/countries^10^,^49^,^50^, environmental differences could explain the discrepancies of previous finding with the present study. Future studies, especially in different contexts, are necessary to clarify such alternative causalities. The results concerning migrant density deserve further discussion. At an individual-level, there is strong evidence for a higher incidence and prevalence of NAPD among migrants^51^,^52^, including in France^49^,^53^. Several reports studied the influence of ethnic density. Boydell *et al.* found that incidence rate ratios (IRR) of schizophrenia in non-white ethnic minorities in South-London significantly increased as the proportion of such minorities in the local population decreased^5^. A prevalence-based study by Termorshuizen *et al.* in Utrecht, also at an individual-level, is also consistent with this “ethnic density effect”. Although the rate ratio of NAPD among ethnic minorities compared with native populations was significantly increased in all the studied neighbourhoods, there were significant individual variations according to the neighbourhood. As for incidence, this rate ratio decreased with increasing minority density. Moreover, the RR was higher for Dutch natives living in a high non-Dutch density neighbourhood^18^. Interestingly, in the present report, at an ecological-level, the migrant density was not associated with the distribution of cases. This could be explained by a lower incidences of NAPD among migrants living in high migrant density settings. Additional explanations may also be proposed. Firstly, in this study, we considered only the first-generation migrants; and previous incidence and prevalence-based reports studied at least two generations, even three sometimes^49^,^52^. Moreover, the use of census data could underestimate the migrant population (e.g. undocumented, or recently moved)^52^, particularly in economically deprived areas. These two facts may minimize the influence of migrant density, leading to a measurement bias. Secondly, migrant status could also represent a modifier factor and explain an absence of association at an area-level. For instance, some migrants with NAPD, experiencing chronic social defeat and poor quality of life, could go back to their native country. This “selective return” to native country, which could be a mirror image of the Ødegaard’s “selective migration” hypothesis^54^, may bias prevalence-based analyses. Last but not least, migrants with NAPD could have a shorter duration of disease, as was the case for African-Caribbeans in England^55^. To help decide between different possible explanations, further studies, using different methodology–in particular multilevel analyses–are necessary.

### Limitations

Several limitations have to be acknowledged. First, a potential lack of statistical power, which precludes definitive conclusions concerning the influence of migrant density and social fragmentation. However, the necessary statistical power for such ecological studies is difficult to model. In the present study, the dependent variable (number of cases per area) is studied in 53 IRIS. In comparison, in previous ecological studies, such as in the Swedish study of Lögdberg *et al.*, analyses were carried out in 87 communities^56^; the Irish study by Scully *et al.* was carried out in 39 district divisions^38^; and the Spanish study by Moreno *et al.* conducted in 80 municipalities^24^. Second, our data came from an 8-week prevalence of treated patients. While several methods allowed us to estimate the number of potentially missed cases (estimated to more than 20%^27^), we could not analyse them geographically, because we were not able to locate them–except those from the leakage study. Moreover, spatial distribution might also have been biased by healthcare structures in different locations e.g. patients living far from the out patient clinics may have a poorer access to psychiatric care^57^. However, we included data from GPs. Furthermore, as the study concerned treated patients, it might under-represent some clinical profiles, for instance those with lack of insight or milder forms, which could be another selection bias. Our conclusions are therefore limited to treated subjects. Nevertheless, most of prevalence and incidence-based studies are based on treated patients; whilst general-population surveys may have other selection biases, such as selective refusal. Finally, the approach used in this study does not allow for conclusions at an individual level.

## Conclusion

This ecological study, using Bayesian methods, found that the distribution, in small areas, of prevalent cases of NAPD patients was associated with economic deprivation. This has implications for the implementation of health care structures in deprived areas. Further studies, particularly in varied environments, will be useful to replicate these findings. Bayesian methods are probably best suited for such studies. When frequentist methods are used, as a minimum requirement, their validity has to be tested and reported. General population studies, based on dimensional measures of psychosis severity or on attenuated psychoses, such as schizotypy^58^, could also inform the relation between psychotic disorders and the environment, avoiding some of the biases associated with studies limited to clinically significant disorders. Moreover, further studies are warranted to assess the involvement of socio-economic settings in the aetiology and course of psychosis. Another important challenge for future research will be to combine multilevel techniques, which allow for cross-level interaction (i.e. between individual and population level) modelling and the Bayesian methods that take spatial correlation into account.

## Additional Information

**How to cite this article**: Pignon, B. *et al.* Spatial distribution of psychotic disorders in an urban area of France: an ecological study. *Sci. Rep.* **6**, 26190; doi: 10.1038/srep26190 (2016).

## Figures and Tables

**Table 1:** Number of cases and prevalences per gender and age-band.

Age-bands	Female	Male	Total
	Number of cases	Number of cases	Number of cases
	Population-at-risk^1^	Population-at-risk^1^	Population-at-risk^1^
	Prevalence (%) [95% CI^2^]	Prevalence (%) [95% CI]	Prevalence (%) [95% CI]
18–24	6	11	17
	7651	6799	14450
	0.07 [0.01–0.15]	0.16 [0.07–0.25]	0.12 [0.07–0.18]
25–39	31	86	117
	15865	14859	30724
	0.20 [0.12–0.26]	0.58 [0.46–0.70]	0.38 [0.31–0.45]
40–54	69	85	154
	12906	11108	24014
	0.53 [0.39–0.67]	0.76 [0.61–0.91]	0.64 [0.54–0.74]
55+	36	34	70
	31752	25953	57705
	0.11 [0.07–0.15]	0.13 [0.09–0.17]	0.12 [0.09–0.15]

^1^Population-at-risk: number of resident aged 18 and over.

^2^CI: Confidence interval.

**Table 2:** Comparison of frequentist models.

Poisson regression model	Akaike information criterion (AIC)	
Basic model (adjusted for age and sex)	292.43	
1 explanatory variable	MIG^1^	289.57
	ECON^2^	285.77
	FRAG^3^	289.95
2 explanatory variables: ECON+	MIG	288.59
	FRAG	286.22
**Negative binomial regression model**	**Akaike information criterion (AIC)**	
Basic model (adjusted for age and sex)	286.29	
1 explanatory variable:	MIG^1^	286.44
	ECON^2^	286.05
	FRAG^3^	285.94
2 explanatory variables: FRAG+	MIG	286.72
	ECON	286.72

^1^MIG: Migrant density: standardized percentage of first generation of migrants (foreign-born or foreigners).

^2^ECON: Economic deprivation: standardized percentage of unemployed and proportion of households not owning (at least) one car.

^3^FRAG: Social fragmentation: standardized proportion of people who had lived in an IRIS for less than 2 years and the proportion of people living alone.

**Table 3:** Comparison of Bayesian models.

		Deviation Information Criterion (DIC)			
Model		IND^4^	IAR^5^	BYM^6^	LER^7^
Basic model (adjusted for age and sex)		270.11	270.91	270.01	269.54
MIG^1^		268.65	269.93	270.05	267.46
ECON^2^		266.03	269.80	269.42	265.12
FRAG^3^		266.54	270.12	271.42	267.56
Two explanatory variables: ECON+:	MIG	269.24	270.08	270.18	265.69
	FRAG	267.08	270.31	270.26	266.67

^1^MIG: Migrant density: standardized percentage of first generation of migrants (foreign-born or foreigners).

^2^ECON: Economic deprivation: standardized percentage of unemployed and proportion of households not owning (at least) one car.

^3^FRAG: Social fragmentation: standardized proportion of people who had lived in an IRIS for less than 2 years and the proportion of people living alone.

^4^IND: independent model.

^5^IAR: intrinsic autoregressive model.

^6^BYM: Besag, York and Molié's model.

^7^LER: Leroux’s model.

## References

[b1] Hart J. On ecological studies: a short communication. Dose-Response Publ. Int. Hormesis Soc. 9, 497–501 (2011). 10.2203/dose-response.10-046.Hart PMC3315170 22461759

[b2] Richardson S., Thomson A., Best N. & Elliott P. Interpreting Posterior Relative Risk Estimates in Disease-Mapping Studies. Environ. Health Perspect. 112, 1016–1025 (2004). 15198922 10.1289/ehp.6740 PMC1247195

[b3] March D. *et al.* Psychosis and place. Epidemiol. Rev. 30, 84–100 (2008). 18669521 10.1093/epirev/mxn006

[b4] Szöke A. *et al.* Rural-urban variation in incidence of psychosis in France: a prospective epidemiologic study in two contrasted catchment areas. BMC Psychiatry 14, 78 (2014). 24636392 10.1186/1471-244X-14-78 PMC3995443

[b5] Boydell J. *et al.* Incidence of schizophrenia in ethnic minorities in London: ecological study into interactions with environment. Br. Med. J. 323, 1336–1338 (2001). 11739218 10.1136/bmj.323.7325.1336 PMC60671

[b6] Veling W. *et al.* Ethnic Density of Neighborhoods and Incidence of Psychotic Disorders Among Immigrants. Am. J. Psychiatry 165, 66–73 (2008). 18086750 10.1176/appi.ajp.2007.07030423

[b7] Lasalvia A. *et al.* First-contact incidence of psychosis in north-eastern Italy: influence of age, gender, immigration and socioeconomic deprivation. Br. J. Psychiatry 205, 127–134 (2014). 24723631 10.1192/bjp.bp.113.134445

[b8] Croudace T. J., Kayne R., Jones P. B. & Harrison G. L. Non-linear relationship between an index of social deprivation, psychiatric admission prevalence and the incidence of psychosis. Psychol. Med. 30, 177–185 (2000). 10722188 10.1017/s0033291799001464

[b9] Kirkbride J. B., Jones P. B., Ullrich S. & Coid J. W. Social deprivation, inequality, and the neighborhood-level incidence of psychotic syndromes in East London. Schizophr. Bull. 40, 169–180 (2014). 23236081 10.1093/schbul/sbs151 PMC3885290

[b10] Allardyce J. *et al.* Social fragmentation, deprivation and urbanicity: relation to first-admission rates for psychoses. Br. J. Psychiatry J. Ment. Sci. 187, 401–406 (2005). 10.1192/bjp.187.5.401 16260813

[b11] Omer S. *et al.* Neighbourhood-level socio-environmental factors and incidence of first episode psychosis by place at onset in rural Ireland: The Cavan–Monaghan First Episode Psychosis Study [CAMFEPS]. Schizophr. Res. 152, 152–157 (2014). 24342585 10.1016/j.schres.2013.11.019 PMC3906531

[b12] Bhavsar V., Boydell J., Murray R. & Power P. Identifying aspects of neighbourhood deprivation associated with increased incidence of schizophrenia. Schizophr. Res. 156, 115–121 (2014). 24731617 10.1016/j.schres.2014.03.014

[b13] Stolk R. P. *et al.* Universal risk factors for multifactorial diseases. Eur. J. Epidemiol. 23, 67–74 (2007). 18075776 10.1007/s10654-007-9204-4

[b14] Grimes D. A. & Schulz K. F. Descriptive studies: what they can and cannot do. The Lancet 359, 145–149 (2002). 10.1016/S0140-6736(02)07373-7 11809274

[b15] van Os J., Hanssen M., Bijl R. & Vollebergh W. Prevalence of psychotic disorder and community level of psychotic symptoms: An urban-rural comparison. Arch. Gen. Psychiatry 58, 663–668 (2001). 11448373 10.1001/archpsyc.58.7.663

[b16] Saha S., Chant D., Welham J. & McGrath J. A systematic review of the prevalence of schizophrenia. PLoS Med. 2, e141 (2005). 15916472 10.1371/journal.pmed.0020141 PMC1140952

[b17] Tizón J. L. *et al.* Neighborhood differences in psychoses: prevalence of psychotic disorders in two socially-differentiated metropolitan areas of Barcelona. Schizophr. Res. 112, 143–148 (2009). 19411159 10.1016/j.schres.2009.04.008

[b18] Termorshuizen F., Smeets H. M., Braam A. W. & Veling W. Neighborhood ethnic density and psychotic disorders among ethnic minority groups in Utrecht City. Soc. Psychiatry Psychiatr. Epidemiol. 49, 1093–1102 (2014). 24554124 10.1007/s00127-014-0842-z

[b19] Lee D. CARBayes: An R Package for Bayesian Spatial Modeling with Conditional Autoregressive Priors. J. Stat. Softw. 55, 1–24 (2013).

[b20] Bertolino F., Racugno W. & Moreno E. Bayesian Model Selection Approach to Analysis of Variance Under Heteroscedasticity. J. R. Stat. Soc. Ser. Stat. 49, 495–502 (2000).

[b21] Cameron A. C. & Trivedi P. K. Regression Analysis of Count Data. (Cambridge University Press, 2013).

[b22] Torabi M. Spatial modeling using frequentist approach for disease mapping. J. Appl. Stat. 39, 2431–2439 (2012).

[b23] Banerjee S., Carlin B. P. & Gelfand A. E. In Hierarchical Modeling and Analysis for Spatial Data 73–96 (Chapman and Hall/CRC, 2014).

[b24] Moreno B., García-Alonso C. R., Negrín Hernández M. A., Torres-González F. & Salvador-Carulla L. Spatial analysis to identify hotspots of prevalence of schizophrenia. Soc. Psychiatry Psychiatr. Epidemiol. 43, 782–791 (2008). 18500483 10.1007/s00127-008-0368-3

[b25] French National Institute for Statistics and Economic Studies. Definition of IRIS. Available at: http://www.insee.fr/en/methodes/default.asp? page=definitions/iris.htm. Date of access: 09/03/2016 (2015).

[b26] INSEE. French National Institute for Statistics and Economic Studies (Institut National de la Statistique et des Études Économiques: INSEE). Available at: http://www.insee.fr/en/default.asp. Date of access: 09/03/2016 (2016).

[b27] Szöke A. *et al.* Prevalence of psychotic disorders in an urban area of France. BMC Psychiatry 15, 204 (2015). 10.1186/s12888-015-0588-5 PMC4548685 26303009

[b28] American Psychiatric Association. *DSM-IV-TR: Diagnostic and Statistical Manual of Mental Disorders. (The Association, 2000)*.

[b29] Aleman A., Kahn R. S. & Selten J.-P. Sex differences in the risk of schizophrenia: evidence from meta-analysis. Arch. Gen. Psychiatry 60, 565–571 (2003). 12796219 10.1001/archpsyc.60.6.565

[b30] Spiegelhalter D. J., Best N. G., Carlin B. P. & Van Der Linde A. Bayesian measures of model complexity and fit. J. R. Stat. Soc. Ser. B Stat. Methodol. 64, 583–639 (2002).

[b31] Guo H.-R. Age adjustment in ecological studies: using a study on arsenic ingestion and bladder cancer as an example. BMC Public Health 11, 820 (2011). 22014275 10.1186/1471-2458-11-820 PMC3224075

[b32] Allardyce J. & Boydell J. Review: the wider social environment and schizophrenia. Schizophr. Bull. 32, 592–598 (2006). 16849399 10.1093/schbul/sbl008 PMC2632272

[b33] Dean C. B. Testing for Overdispersion in Poisson and Binomial Regression Models. J. Am. Stat. Assoc. 87, 451–457 (1992).

[b34] Cliff A. & Ord K. Testing for Spatial Autocorrelation Among Regression Residuals. Geogr. Anal. 4, 267–284 (1972).

[b35] Leroux B. G., Lei X. & Breslow N. In Statistical Models in Epidemiology, the Environment, and Clinical Trials (*eds.* Halloran M. E. & Berry D. ) 179–191 (Springer: New York, 1999).

[b36] Cowles M. K. & Carlin B. P. Markov Chain Monte Carlo Convergence Diagnostics: A Comparative Review. J. Am. Stat. Assoc. 91, 883–904 (1996).

[b37] Plummer M., Best N., Cowles K. & Vines K. CODA: convergence diagnosis and output analysis for MCMC. R News 6, 7–11 (2006).

[b38] Scully P. J., Owens J. M., Kinsella A. & Waddington J. L. Schizophrenia, schizoaffective and bipolar disorder within an epidemiologically complete, homogeneous population in rural Ireland: small area variation in rate. Schizophr. Res. 67, 143–155 (2004). 14984873 10.1016/S0920-9964(03)00194-4

[b39] Perälä J. *et al.* Geographic variation and sociodemographic characteristics of psychotic disorders in Finland. Schizophr. Res. 106, 337–347 (2008). 18804345 10.1016/j.schres.2008.08.017

[b40] Werner S., Malaspina D. & Rabinowitz J. Socioeconomic Status at Birth is Associated With Risk of Schizophrenia: Population-Based Multilevel Study. Schizophr. Bull. 33, 1373–1378 (2007). 17443013 10.1093/schbul/sbm032 PMC2779876

[b41] O’Donoghue B. *et al.* Neighbourhood characteristics and the incidence of first-episode psychosis and duration of untreated psychosis. Psychol. Med. 46, 1367–1378 (2016). 27032697 10.1017/S003329171500286X

[b42] van Os J. & Kapur S. Schizophrenia. Lancet 374, 635–645 (2009). 19700006 10.1016/S0140-6736(09)60995-8

[b43] Marwaha S. & Johnson S. Schizophrenia and employment-a review. Soc. Psychiatry Psychiatr. Epidemiol. 39, 337–349 (2004). 15133589 10.1007/s00127-004-0762-4

[b44] Ngamini Ngui A. *et al.* Does elapsed time between first diagnosis of schizophrenia and migration between health territories vary by place of residence? A survival analysis approach. Health Place 20, 66–74 (2013). 23376731 10.1016/j.healthplace.2012.12.003

[b45] Kirkbride J. B. Hitting the floor: understanding migration patterns following the first episode of psychosis. Health Place 28, 150–152 (2014). 24845239 10.1016/j.healthplace.2014.04.010 PMC4076512

[b46] Thornicroft G., Brohan E., Rose D., Sartorius N. & Leese M. Global pattern of experienced and anticipated discrimination against people with schizophrenia: a cross-sectional survey. The Lancet 373, 408–415 (2009). 10.1016/S0140-6736(08)61817-6 19162314

[b47] Ellett L., Freeman D. & Garety P. A. The psychological effect of an urban environment on individuals with persecutory delusions: the Camberwell walk study. Schizophr. Res. 99, 77–84 (2008). 18061407 10.1016/j.schres.2007.10.027

[b48] Lindert J., Schouler-Ocak M., Heinz A. & Priebe S. Mental health, health care utilisation of migrants in Europe. Eur. Psychiatry 23, Supplement 1, 14–20 (2008). 18371575 10.1016/S0924-9338(08)70057-9

[b49] Amad A. *et al.* Increased prevalence of psychotic disorders among third-generation migrants: results from the French Mental Health in General Population survey. Schizophr. Res. 147, 193–195 (2013). 23570896 10.1016/j.schres.2013.03.011

[b50] Selten J.-P., Laan W., Kupka R., Smeets H. M. & van Os J. Risk of psychiatric treatment for mood disorders and psychotic disorders among migrants and Dutch nationals in Utrecht, The Netherlands. Soc. Psychiatry Psychiatr. Epidemiol. 47, 271–278 (2012). 21203744 10.1007/s00127-010-0335-7

[b51] Cantor-Graae E. & Selten J.-P. Schizophrenia and migration: a meta-analysis and review. Am. J. Psychiatry 162, 12–24 (2005). 15625195 10.1176/appi.ajp.162.1.12

[b52] Bourque F., van der Ven E. & Malla A. A meta-analysis of the risk for psychotic disorders among first- and second-generation immigrants. Psychol. Med. 41, 897–910 (2011). 20663257 10.1017/S0033291710001406

[b53] Tortelli A. *et al.* Different rates of first admissions for psychosis in migrant groups in Paris. Soc. Psychiatry Psychiatr. Epidemiol. 49, 1103–1109 (2013). 24270936 10.1007/s00127-013-0795-7 PMC4283097

[b54] Ødegaard Ö. Emigration and insanity. Acta Psychiatr. Neurol. Scand. *Suppl.* **4**, 1–206 (1932).

[b55] McKenzie K. *et al.* Comparison of the outcome and treatment of psychosis in people of Caribbean origin living in the UK and British Whites. Report from the UK700 trial. Br. J. Psychiatry 178, 160–165 (2001). 11157430 10.1192/bjp.178.2.160

[b56] Lögdberg B., Nilsson L.-L., Levander M. T. & Levander S. Schizophrenia, neighbourhood, and crime. Acta Psychiatr. Scand. 110, 92–97 (2004). 15233709 10.1111/j.1600-0047.2004.00322.x

[b57] Maylath E., Seidel J., Werner B. & Schlattmann P. Geographical analysis of the risk of psychiatric hospitalization in Hamburg from 1988–1994. Eur. Psychiatry 14, 414–425 (1999). 10683627 10.1016/s0924-9338(99)00226-6

[b58] Szöke A., Kirkbride J. B. & Schürhoff F. Universal prevention of schizophrenia and surrogate endpoints at population level. Soc. Psychiatry Psychiatr. Epidemiol. 49, 1347–1351 (2014). 24488181 10.1007/s00127-014-0829-9

